# An intra-cytoplasmic route for SARS-CoV-2 transmission unveiled by Helium-ion microscopy

**DOI:** 10.1038/s41598-022-07867-0

**Published:** 2022-03-08

**Authors:** Antonio Merolli, Leila Kasaei, Santhamani Ramasamy, Afsal Kolloli, Ranjeet Kumar, Selvakumar Subbian, Leonard C. Feldman

**Affiliations:** 1 Department of Physics and Astronomy, School of Arts and Sciences, Rutgers University, 136 Frelinghuysen Road, Piscataway, NJ 08854 USA; 2 Public Health Research Institute (PHRI), New Jersey Medical School, Rutgers University, 225 Warren Street, Newark, NJ 07103 USA; 3 Department Physics and Astronomy, Rutgers University, DLS Building, 145 Bevier Road, Room 108, Piscataway, NJ 08854 USA

**Keywords:** SARS-CoV-2, Cell signalling

## Abstract

SARS-CoV-2 virions enter the host cells by docking their spike glycoproteins to the membrane-bound Angiotensin Converting Enzyme 2. After intracellular assembly, the newly formed virions are released from the infected cells to propagate the infection, using the extra-cytoplasmic ACE2 docking mechanism. However, the molecular events underpinning SARS-CoV-2 transmission between host cells are not fully understood. Here, we report the findings of a scanning Helium-ion microscopy study performed on Vero E6 cells infected with mNeonGreen-expressing SARS-CoV-2. Our data reveal, with unprecedented resolution, the presence of: (1) long tunneling nanotubes that connect two or more host cells over submillimeter distances; (2) large scale multiple cell fusion events (syncytia); and (3) abundant extracellular vesicles of various sizes. Taken together, these ultrastructural features describe a novel intra-cytoplasmic connection among SARS-CoV-2 infected cells that may act as an alternative route of viral transmission, disengaged from the well-known extra-cytoplasmic ACE2 docking mechanism. Such route may explain the elusiveness of SARS-CoV-2 to survive from the immune surveillance of the infected host.

## Introduction

In the well-documented route of SARS-CoV-2 infection of the upper respiratory tract, virions contained in airborne droplets enter the cells of the respiratory epithelium by docking their spike glycoproteins to the membrane-bound angiotensin converting enzyme 2 (ACE2)^1^. Subsequently, the infection can spread downwards the respiratory tree until it finally reaches the surfactant-producing alveolar type 2 pneumocytes. Successful infection of the lung cells leads to severe damage in gas exchange (O_2_ uptake and CO_2_ release), which provokes the dreadful symptomatology characterized by shortness of breath. The overall alveolar damage associated with progressive SARS-CoV-2 infection is difficult to treat and can lead to death^2,3^. Mechanisms of SARS-CoV-2 infection and tissue damage are studied in clinical patients^1^, animal models^4^ and in vitro models^5^. High-resolution imaging studies of alterations at the cellular level are critical to a complete understanding of the mechanisms of SARS-CoV-2 infection. We are presently exploiting the specific capabilities of scanning Helium-ion microscopy (HeIM) to study host–pathogen interactions in these three environments.

Helium-ion microscopy can image biological samples at nanometer resolution^6^ after a minimal processing (often a formalin-fixation step) or even no processing (although SARS-CoV-2 infected samples require inactivation prior to imaging, for safety reasons). Due to its charge neutralization capability, HeIM can image biological samples (an insulating material) without the need of the electroconductive coating typically required by Scanning Electron Microscopy (SEM)^6^. A comprehensive review on the subject of bioimaging with HeIM has recently been published by Schmidt et al.^7^. This “no coating” capability of HelM represents a great advantage as such coatings, even though only a few nanometers thick, can significantly alter and conceal fine details of biological structures. Literature already provides photomicrographs of SARS-CoV-2 infected Vero E6 cells cultures as they appear using SEM with an electroconductive coating^8^ or using HeIM without^9^.

Coronaviruses are enveloped viruses, surrounded by a host membrane acquired during the budding of virions through the host endoplasmic reticulum/Golgi apparatus. When the newly formed virions are released outside the cell, they propagate the infection using the ACE2 docking mechanism. Fresh bud virions fuse their membrane with the one of the target cell and then release the viral genomic RNA inside the cytoplasm. The replication of new viruses “highjacks” several components of the intracellular vesicles machinery (newly replicated intracellular coronaviruses are found inside larger vesicular/vacuolar structures; they are not free as single virions inside the cytoplasm^10^). At this point, humoral and cellular immunity (antibodies and T-lymphocytes), and several other innate immune mechanisms (e.g., interferons and surfactant proteins), can interfere with this extra-cytoplasmic mechanism of infection. For example, the host immune system can neutralize the virus and eventually extinguish the infection. Indeed, several intervention strategies to control SARS-CoV-2 infection target the host immune defense to extra-cytoplasmic virions, like vaccination (which boosts antibody formation)^11^; convalescent plasma administration (which provides a high titer of neutralizing antibodies)^12^; laboratory-produced “cocktails” of neutralizing antibodies^13^. Unfortunately, several patients develop a hyper-acute inflammatory response, some of them on an autoimmune base^14^, which can contribute to disease progression even in the absence of virions. This response can lead to a fatal multiorgan failure and subsequent death. Although therapeutic options are available, like corticosteroids^15^ or cytokines adsorption^16^, they cannot always control this often fatal progression of the coronavirus disease (COVID19). Despite a large percentage of the population in several countries being vaccinated, the occurrence of new symptomatic infections in individuals who were effectively vaccinated (“breakthrough infections” in vaccine-responders) has raised the question as to the cause of immune escape and renewed disease progression by SARS-CoV-2^17^. Vaccination significantly reduces the severity of infections, but a fraction of vaccinated individuals are re-infected with SARS-CoV-2 that can progress to an extremely severe illness and possible death^18^. Importantly, the viral entry mechanisms into the host cells are not fully understood and the question of how the virus transmits, infects and propagates in a host with robust immune response remains unanswered. In this study, we applied HelM to interrogate the altered morphology of Vero E6 cells infected with mNeonGreen-expressing SARS-CoV-2. We demonstrate the presence of: (1) long tunneling nanotubes (TNT) which strongly connect two or more cells over submillimeter distances; (2) large scale multiple cell fusion events (syncytia); and (3) abundant extracellular vesicles of various sizes, with unprecedented resolution. Based on the findings, we propose that these three ultrastructural features describe a fully intra-cytoplasmic connection among cells that may act as an alternative route of viral transmission and infection, disengaged from the conventional extra-cytoplasmic ACE2 docking mechanism. Futhermore, the intracytoplasmic viral transmission may explain the ability of SARS-CoV-2 to escape the immune surveillance and the host response.

## Results

### Inter-cellular connections by tunneling nano tubes

**Figure 1:**
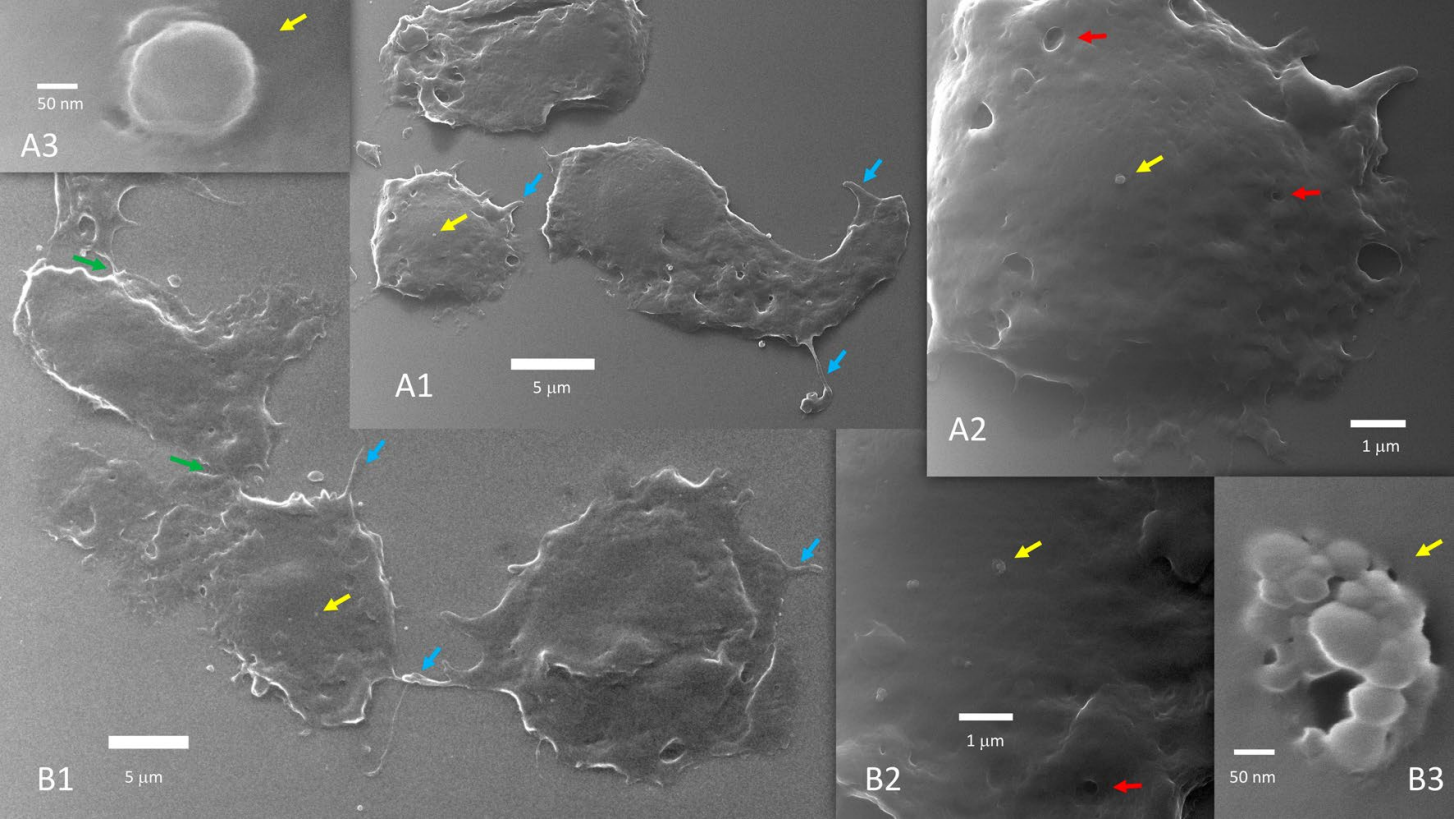
Uninfected Vero E6 cells (**A1**, **A2**, **A3** and **B1**, **B2**, **B3** are progressive zoom-in of two different group of cells). Uninfected cells do not fuse during growth and when progressing toward confluency. Coarse filopodia (blue arrows) and caveolae (red arrows) are present in these cells. There are bridge points of contact (green arrows). Vesicles can be seen over the cell surface (yellow arrows) or close to the cells. In (**A3**), a 200 nm vesicle is seen budding or merging at the cell membrane level. (**B3**) Likely captured the exosomes discharge from a multivesicular body.

It has been reported that SARS-CoV-2 infected Vero E6 cells produce many filopodia, and they are longer than those present in uninfected cells^19,20^. We observed these very long filopodia and followed their path. We confirmed that such long filopodia are not present in uninfected cells (Fig. 1).

**Figure 2:**
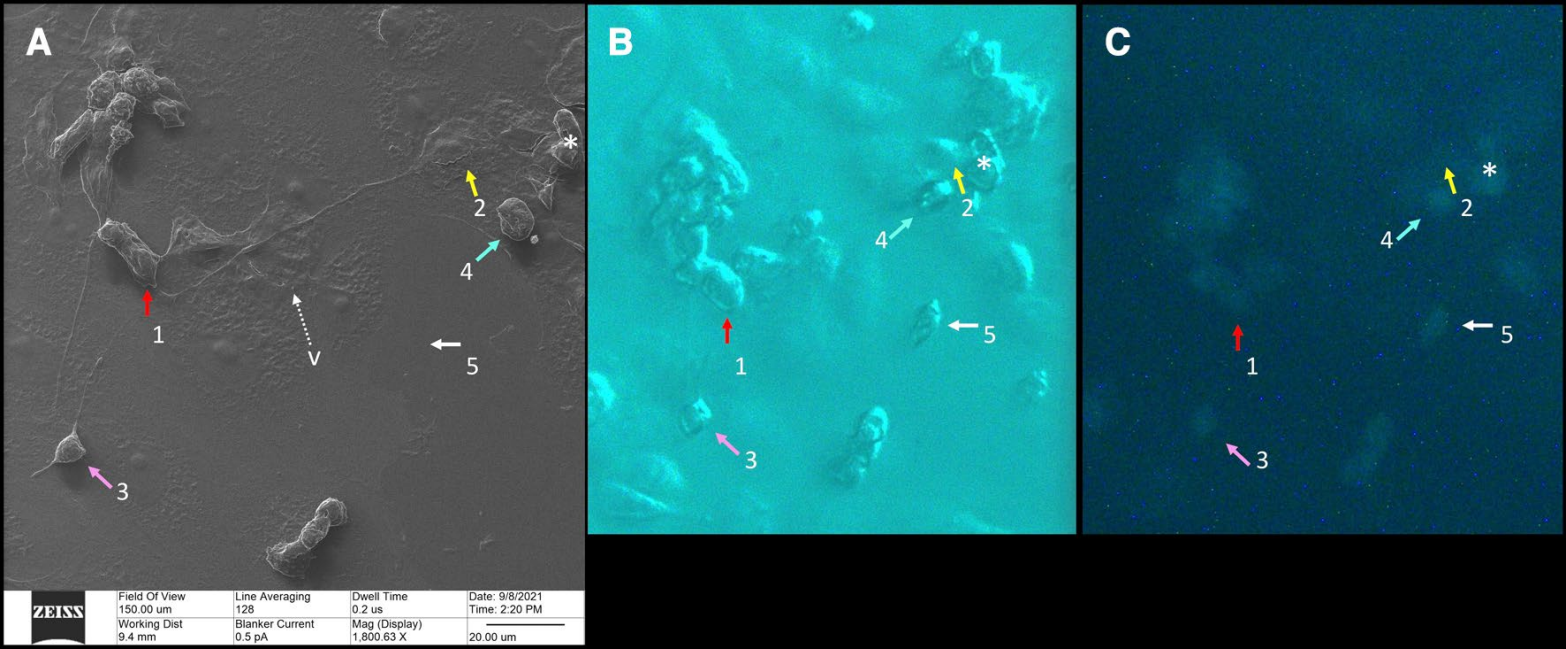
Correlative microscopy. A 150-micron square area has been imaged by HeIM (**A**), phase-contrast microscopy (**B**) and fluorescence microscopy (**C**). Our usual sequence of acquisition is to take a phase-contrast image first, then a fluorescence image, than a HeIM image, all on the same area. HeIM modality, by avoiding electroconductive coating, can allow changes in this sequence when needed. HeIM (**A**) and phase-contrast (**B**) modalities show the morphology of the cells. Fluorescence microscopy (**C**) highlights their infectious status, because only infected cells fluoresce in green. In this area, HeIM (**A**) shows that two cells (1 and 2) are connected by a TNT about 100 microns long. There is also a rounded bipolar cell (3) emitting TNT, and correlative microscopy shows that it is infected; this cell (3) did not change its position regarding the prior acquired phase-contrast photomicrograph. Rounded infected cells, however, tend to detach from the floor; correlation between Phase Contrast (**B**) and HeIM (**A**) shows that cell 4 changed its position slightly and cell 5 moved out of the field. Only HeIM was able to pick-up the presence of vesicles in the area; the tiny one indicated by “v” in (**A**) will be shown at higher magnification in Fig. 8D (HeIM (**A**) × 1800; Optical Microscopy (**B,C**) × 400).

**Figure 3:**
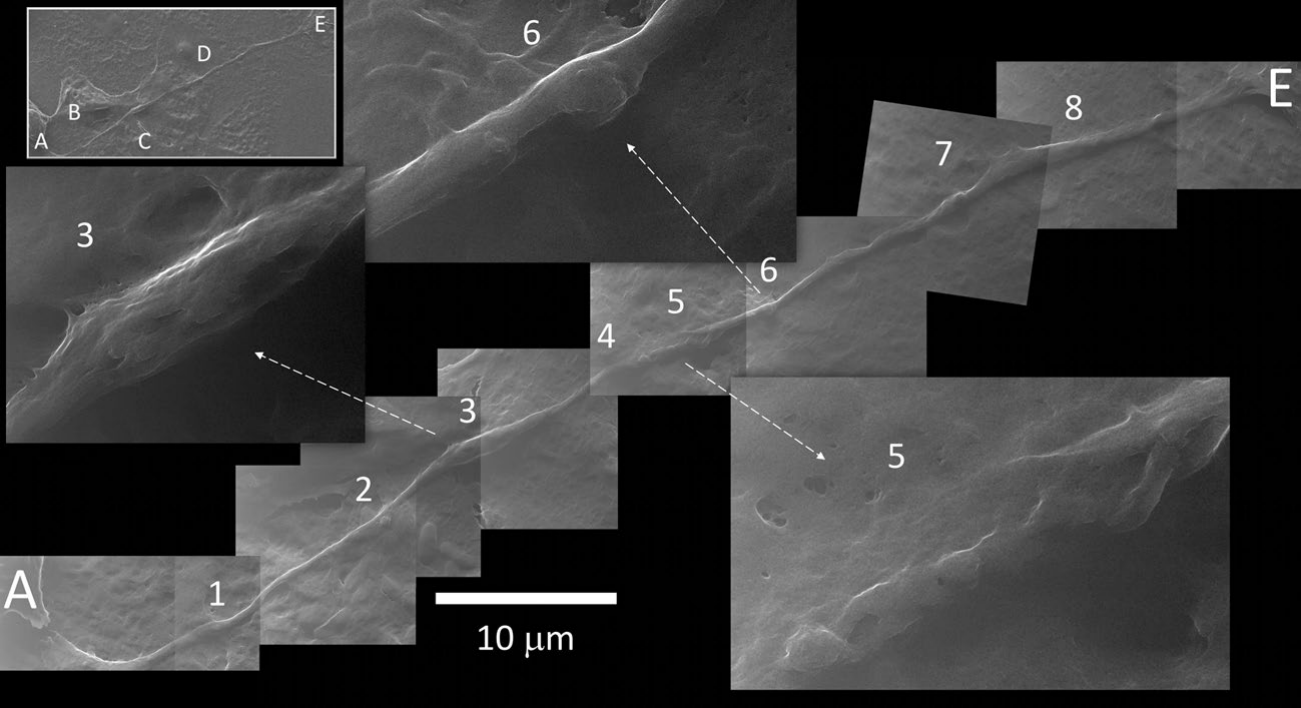
Tunneling nanotube. The long TNT that connects cell A to B (named 1 and 2 in Fig. 2) interacts with at least three other cells along its path (B, C and D, in the top left inset). A mosaic reconstruction of HeIM micrographs (27,000×) highlights 8 notable events, some of which are magnified at a higher definition (×54,000). (1) Spherical bulging; (2) well defined spherical bulging and membrane fusion, in a region rich in mitochondria; (3) diameter enlargement by cargo content; (4) spherical bulging close to a (5) membrane fusion area; (6) well defined spherical bulging; (7) two close spherical bulging; (8) diameter enlargement. Higher definition micrographs show longitudinal cytoskeletal features under the membrane.

These filopodia are visualized even at nanometer diameters using HelM, providing high-resolution imaging of their surface details. It would be difficult to observe these finer details with conventional optical microscopy. Based on our observations, these very long filopodia showed the characteristics of Tunneling Nano Tubes (TNT) (see “Discussion” for the terms “cytonemes” and “TNT” and how they differ from filopodia). These TNT connect two or more infected host cells over submillimeter distances (Fig. 2). They often exceed 40 microns in length and span distances which encompass several cells (Fig. 3).

**Figure 4:**
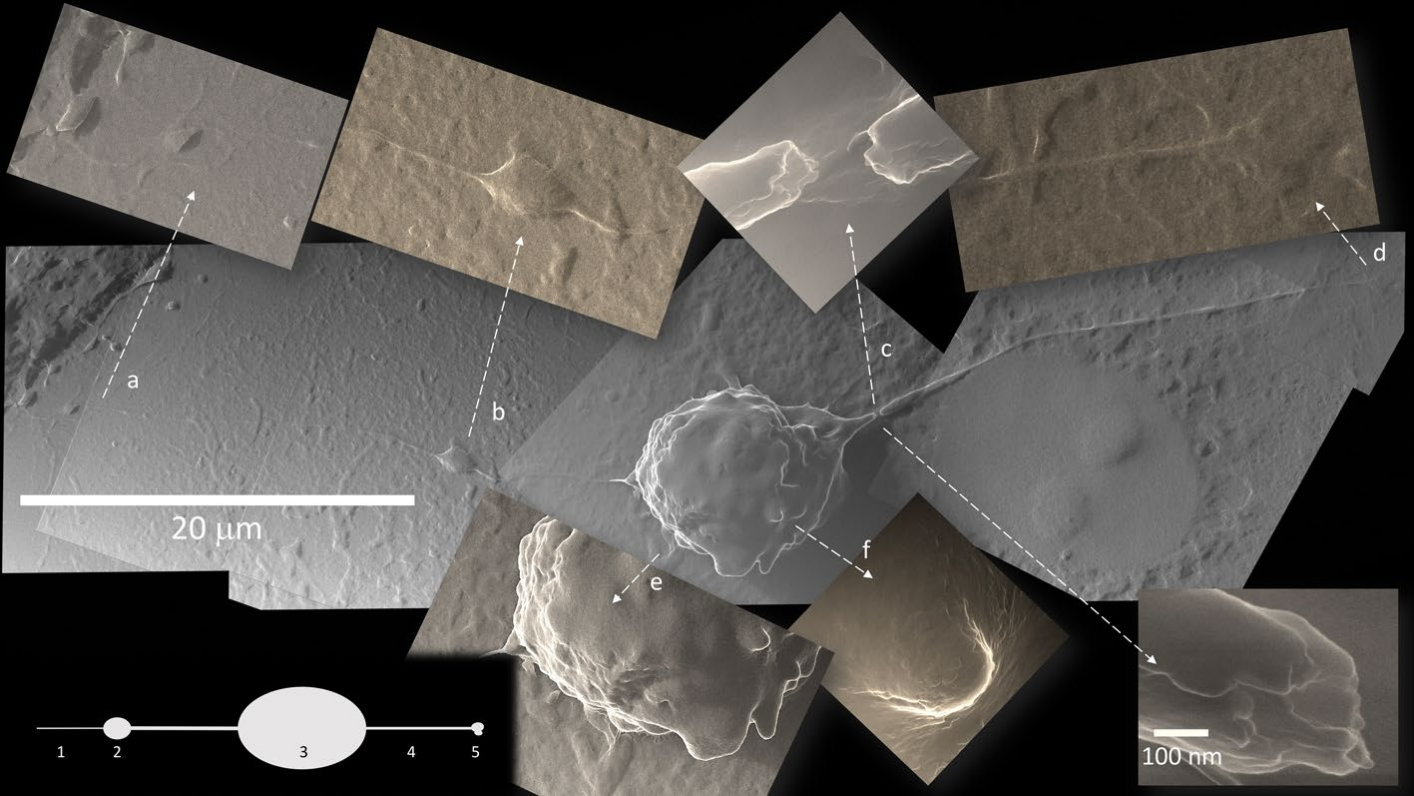
TNT from a polarized infected cell. Some infected cells start to bulge and detach from the floor, while emitting TNT in a specific bipolar fashion. An asymmetry in these bipolar cells is often noted, so that there is a growth cone (b, 2) on one side, that may emit a slight prolongation of variable length (a, 1), while the opposite TNT (4) ends with a polymorphic bulge (d, 5). Breakages at TNT origin close to the cell body visualize an inner content of cylindrical structures of about 25 nm in diameter, suggestive of microtubules (c). A corrugated surface (e) and apoptotic bodies (f) are specific to this infected cell stage (All photomicrographs at 10,800×; apart from c top, 135,000× and c bottom 270,000×).

They generally start with a “growth cone” of specific morphology (Fig. 4); a smaller diameter tube is sometimes observed stemming from it (Fig. 4). Some infected cells bulge and detach from the floor, while emitting TNT in a specific asymmetric bipolar fashion (Supplementary Information S1, S3).

**Figure 5:**
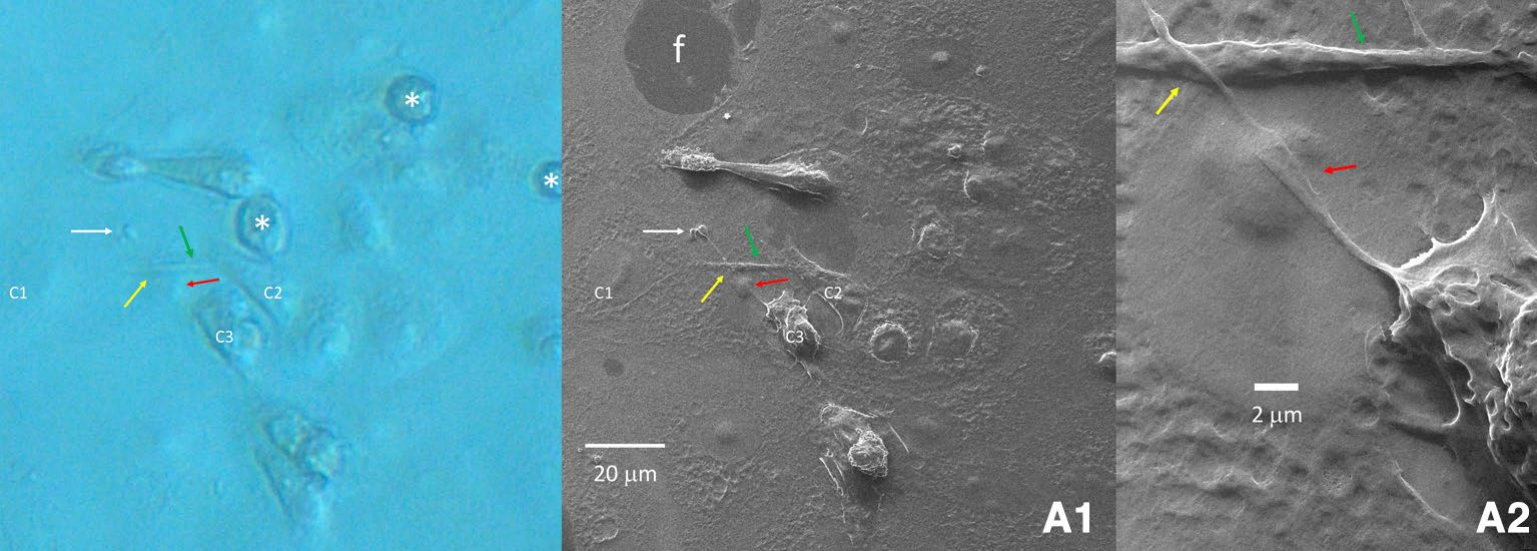
TNT overlap. An area of 130-micron square has been imaged (**A1**) that shows a TNT originating from cell C3 overlapping another TNT which connects cells C1 and C2 (green arrow). A region shows the bare floor of the culture well (f). TNT keep their individual structure when they cross each other (yellow arrow), but they may fuse their membrane when they overlap a cell (red arrow) (magnification in (**A2**)). The specific morphology often seen in a TNT polymorphic bulged termination is pointed by a white arrow. Correlation with a phase contract image taken before HeIM (left) shows three rounded infected cells detached from the floor (asterisks) that were no longer present in the subsequent HeIM photomicrograph.

**Figure 6:**
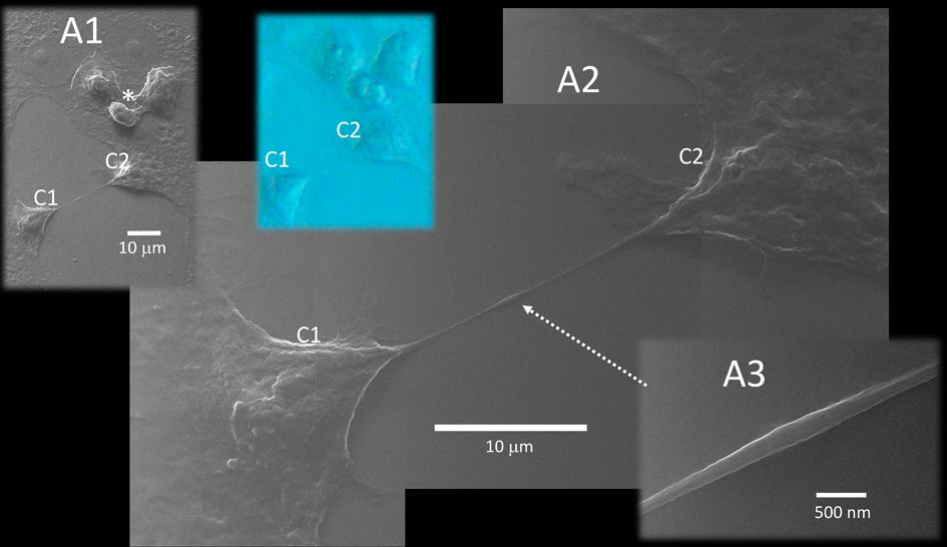
TNT stretched between two host cells. A TNT under mechanical tension between two cells (C1 and C2) lies on the well floor. Fully flat healthy cells and a trio of bulging and detaching infected cells (asterisk in A1) can be seen nearby. This stretched TNT pulls on both cells, shaping them with a stretched morphology (A2). We ruled out the possibility that this tension could be caused by the dehydration process because correlative microscopy showed the same stretched morphology in the hydration state in Phase Contrast prior to HeIM (blue inset). Stretched TNT between two cells often present a bulging mid-way (dotted arrow; magnified in A3) (magnification in A1 × 2700; in A2 × 10,800; in A3 × 77,000).

These TNT keep their individual structure when they cross other TNT, but they may fuse their membrane when overlapping with another cell (Fig. 5). Structures resembling vesicles or mitochondria have been imaged at this fusion point of entry/exit with the cell (Fig. 3). Some TNTs appear under mechanical tension between two cells. In these cases, we documented TNT pulling on both cells resulting in a stretched morphology. We ruled out the possibility that this tension could be caused by the dehydration process because correlative microscopy showed the same stretched morphology in the hydration state in phase-contrast microscopy prior to HeIM. Stretched TNT between two cells often presents a bulging mid-way (Fig. 6). However, the dehydration process likely caused breakage at the TNT origin close to the cell body; when this occurred, we visualized an inner content of cylindrical structures of about 25 nm in diameter (Fig. 4), suggestive of microtubules. TNT in their larger diameter showed similar longitudinal cytoskeletal features underlining the membrane, and they showed regions of bulging content (Fig. 3): this feature is reminiscent of pictures of axonal transport. There were also clear images of spherical bulging along the length of TNT (Fig. 3). Notably, we could visualize regions of 250 nm^2^ or larger, where several cells showed to be connected either by TNT or by fusion (membrane continuity) and, at the same time, extracellular vesicles were present all around, meaning that these three features may co-exist together in the same cell (Fig. 2).

### Cell fusion

**Figure 7:**
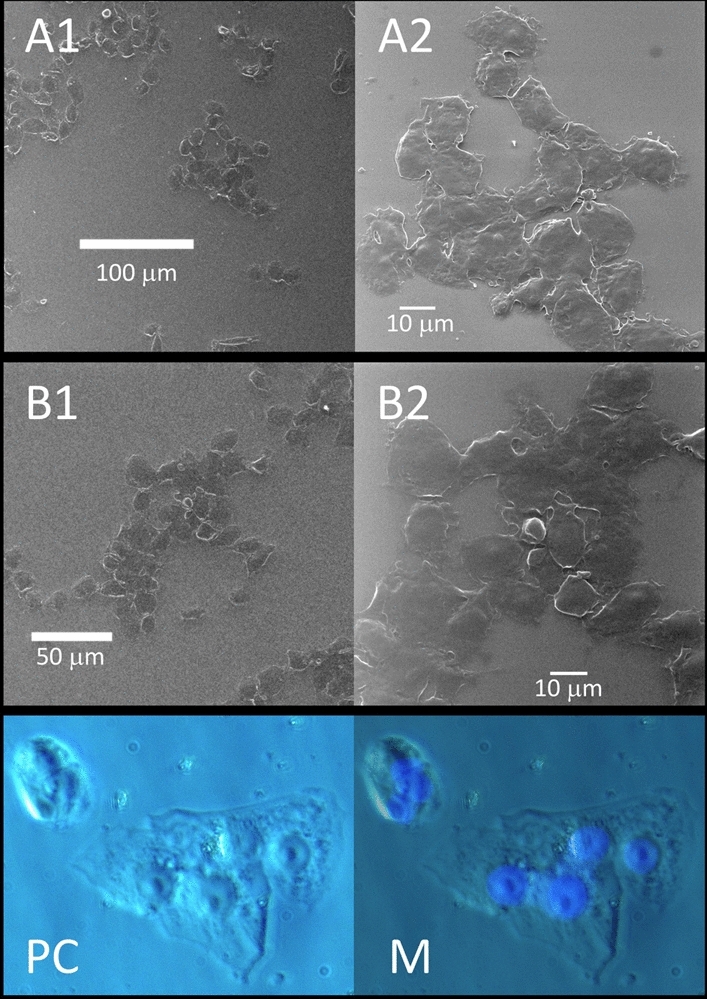
Fusion of SARS-CoV-2 infected host cells. Infected cells form submillimeter clusters of multiple fused cells at 24 h post-infection. HeIM highlights the continuity of their membranes, while fluorescence microscopy shows the presence of multiple DAPI stained fluorescent nuclei (blue in M) in clusters of cells imaged with phase-contrast microscopy (PC).

The virus propagating within Vero E6 cells causes severe cytopathic effect (CPE)^5^. It is possible that the uninfected cells that lie flat over the well-floor, can swell and detach from the floor, may fuse together and may form TNT. We identified that SARS-CoV-2 infected Vero E6 cells can form large-scale (submillimeter) clusters of multiple fused cells (syncytia) at 24 h post-infection. This type of fusion is not observed in uninfected cells. HeIM provided images of the homogeneous continuity of the membrane of the syncytia (Fig. 7A,B). Correlative microscopy showed the presence of multiple nuclei, identified by their specific fluorescent marker DAPI, in these clusters of cells (Fig. 7).

### Extracellular vesicles

**Figure 8:**
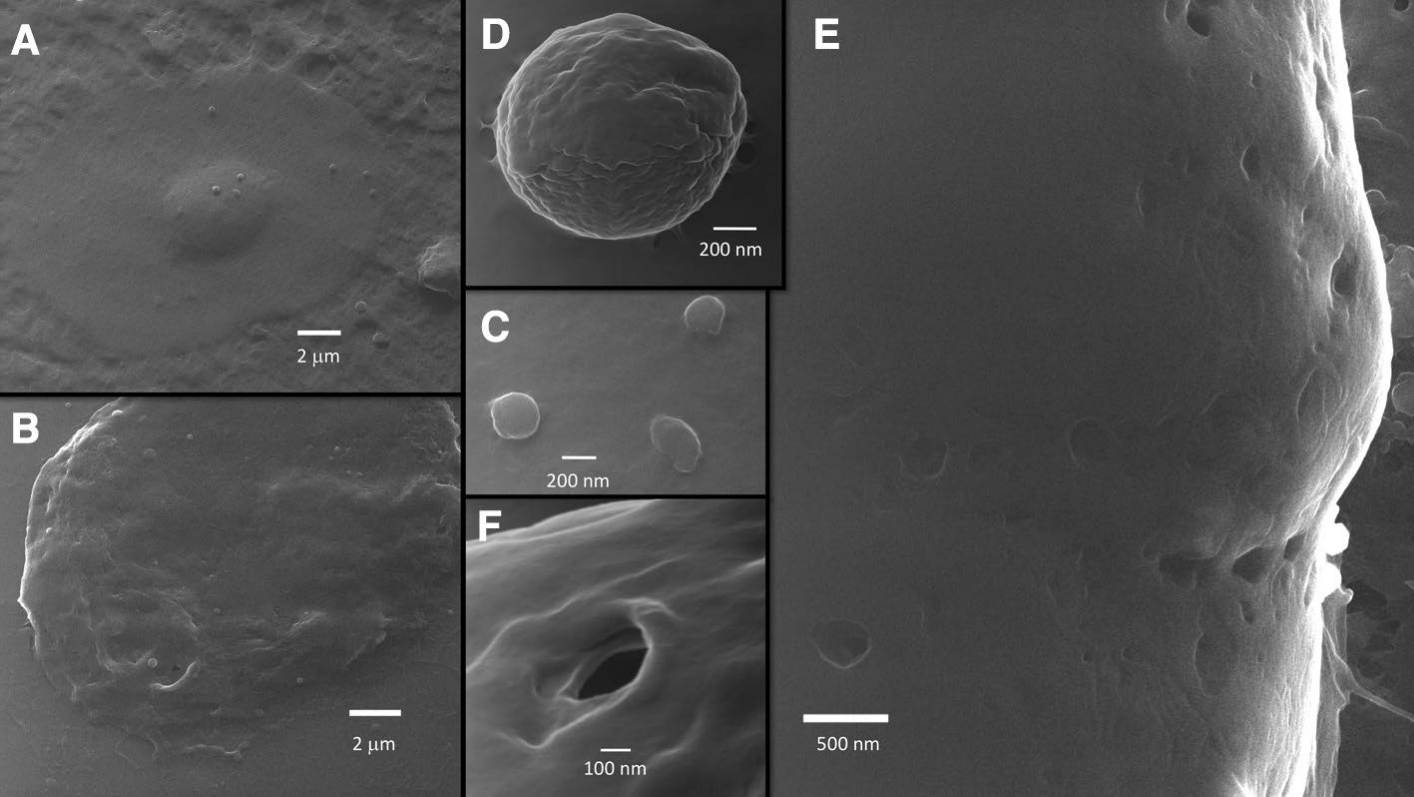
Vesicles and caveolae in SARS-CoV-2 infected cells. Vesicles and caveolae are seen in infected cells at various magnifications (magnification in (**A,B**) × 10,800; (**B,C**) × 135,000; (**D**) × 180,000; (**E**) × 135,000; (**F**) × 270,000).

Many spherical vesicles whose diameter ranged from about 30 nm to more than 1 micron have been observed both in SARS-CoV-2 infected and uninfected Vero E6 cells, apparently in similar numbers (Figs. 1, 8). We documented the extracellular vesicles when they bud from the cell membrane (Figs. 1A3,B3, 8D), or while they are in contact with the membrane (Fig. 8A,C), or as free objects in the extracellular space (Fig. 1C). Apoptotic bodies were observed budding from infected cells (Fig. 8D); some of them were captured just before leaving a disintegrating cell (Supplementary Information S2). A finding that can be put in relation to the presence of extracellular vesicles is that both infected and uninfected VeroE6 cells showed a significant presence of caveolae (Figs. 1, 8E,F). These “holes” in the cell membrane have been previously observed in not-electroconductive-coated mammalian cells; they are the circular aperture of larger spherical cavities formed by the scaffolding “caveolins” proteins^21^. Prior studies using HelM have reported the caveolae as lipid nanodomains^22^. Importantly, caveolae can be both entry or exit points for vesicles in the diameter range of the SARS-CoV-2^23,24^.

## Discussion

### TNT-mediated inter-cellular connections

TNTs are tubular structures of nanometer-to-micrometer diameters that connect the cytoplasm of adjacent or distant cells, thus providing an intracytoplasmic passage to exchange and/or transport biomolecules as small as ions, lipids, nucleic acids, microRNA, cytoplasmic proteins, or as big as whole organelles, such as endosomes, lysosomes, mitochondria and portions of the endoplasmic reticulum or Golgi apparatus. They can be very long, notably over submillimeter distances (spanning several cell diameters)^25–32^. TNTs are reported to be transient structures and can form or disintegrate in a matter of a minutes^25^. They are known to promote the spread of various pathogens, including viruses^33,34^, prions^35^, fungi^36^ and mycoplasma^37^.

TNT were initially hypothesized as a subset of filopodia, but accumulating evidence suggests that TNT are categorically different from filopodia, both in length (longer than filopodia), diameter (as thin as filopodia but as large as axons), composition and function. Unlike filopodia, TNT are capable of mediating vesicular transport. Filopodia are made of F-actin, while TNT, despite being composed of F-actin in their vast majority, can incorporate microtubules too, or be mainly composed of microtubules^38^. Some authors have named long-filopodial bridges containing F-actin as “cytonemes” and differentiated them from TNT^39–41^. We recognize that TNT is now the most accepted term in the literature, even if there are structural variations among them that may justify a further sub-classification. With their growth cone, length, potential microtubule skeleton, and intraluminal transport, TNT resemble the neuronal axons^42^. TNTs may form gap-like junctions between two connected cells over a short distance and they can extend when the two connected cells migrate apart. We documented this mechanical stretch, the capability of TNT to withstand it, and the presence of bulging(s) mid-way. While it is possible that this bulging represents a fusion of two growth cones originating from different cells, or some other phenomenon associated with intraluminal cargo transport, it is also likely that the bulging is just a physical response to the stretching and thinning of the nanotube^43^. TNT may keep its structure when overlapping cells are present in between the two distant cells that they connect. However, we documented the occurrence of possible membrane fusion of overlapping TNT with cells in between, thus suggesting the possibility that the cytoplasm of multiple cells may be connected through a single TNT. Like in axonal transport, TNT diameter enlarges when a bulky content is transported inside. The spherical morphology of some of these contents has been clearly captured by HeIM, which suggests that the transport of vesicles or viral particles is possible through TNTs.

The role of TNTs in SARS-COV-2 infection has been discussed in two recent papers^28,29^. TNT-mediated intracellular spread can protect the viruses from the circulating immune surveillance and possible viral-neutralization activity present in the extracellular matrix. Intercellular viral spread via TNT avoids virus-cell interactions that may initiate host defense signaling, and mount antiviral responses. Many viruses, such as the influenza virus, human immunodeficiency virus (HIV), and herpes simplex virus (HSV), can evade host immunity and avoid pharmaceutical targeting by using TNT to transmit their genomes to naïve/new cells. Our observation using HeIM, when confirmed by multiple independent sources, may suggest that SARS-CoV-2 should be added to this list of viruses that can transmit and cause infection between host cells through TNT. Consistent with this notion, we did not observe any TNT formation in the uninfected Vero E6 cells.

### Cell fusion

Cell–cell fusion (a.k.a. Syncytia) can be induced by certain types of viral infections, such as HIV, respiratory syncytial virus, and HSV^44^. Syncytia formation has been reported in the literature associated with SARS-COV-2 infection^45–47^, most notably in histopathologic lung sections from patients who died from COVID-19^47^. Another relevant finding is that Vero E6 cells, upon expressing the SARS-CoV-2 spike protein, could form syncytia as long as the ACE2 is present, but they cannot when transfected with SARS-CoV spike protein^46^, implying that cell fusion capability may be specific to SARS-CoV-2. It is reasonable to think that virus-induced cell fusion can facilitate the transfer of the viral genome to the neighboring cells^45^ by sharing cytoplasm between the cells. We observed significant Syncytia events only in the SARS-CoV-2 infected Vero E6 cells and not in the uninfected cells.

### Extracellular vesicles

Cell–cell communication can be mediated by factors released in the intercellular space, such as hormones, cytokines, and other inflammatory mediators. The general term extracellular vesicles (EV) refers to any membrane vesicle released into the extracellular space. An accepted classification defines vesicles generated inside the cell and released into the extracellular space as “exosomes,” (diameter range from 30 to 150 nm). In contrast, vesicles pinched off from the plasma membrane are called “microvesicles” (diameter range from 150 to 1000 nm)^48,49^. Some Authors consider apoptotic bodies (bulk protrusions from dying cells that may end up in extracellular vesicles of 800 nm diameter or larger) as part of the EV family. For a long time, EV were considered as “cellular dust” and did not attract much attention from researchers^49^. However, EV have recently been found to play key roles in cell–cell communication, allowing cells to exchange proteins, lipids and genetic material^23,50^.

Viruses might use EV to infect naïve/new cells^51^. The physical and chemical characteristics of many EV, as well as their biogenesis pathways, resemble those of retroviruses. EV generated by virus-infected cells can incorporate viral proteins and fragments of viral RNA, which is similar to the defective (noninfectious) retroviruses. EV are known to facilitate HIV-1 infection and dissemination; HIV-1 has been reported as “entrapped” in exosome aggregates^52^. “Trojan” exosomes might provide retroviruses the ability to take advantage of the cell-encoded intercellular vesicle traffic^52–54^. HIV-1 exploits the surface properties of the exosomes to facilitate rapid infection of progeny virus, and in so doing, camouflages the virus from immune surveillance. Surrounding itself with exosomes, HIV-1 can accelerate its infection and dissemination^52^. It has been hypothesized that SARS-CoV-2 infected cells can release EV with viral antigens or cargo. EV acting as a “Trojan horse” could explain the re-appearance of the viral RNA in patients recovered from COVID-19^24,55^. EV are involved in SARS-CoV-2 infection^56–58^ and could be used as biomarkers of disease severity^59^. SARS-CoV-2 RNA has been identified in the exosomal cargo samples from patients with COVID-19, but not in healthy subjects, suggesting that the virus might use the endocytosis route to spread infection^60^.

Extracellular vesicles can convey pathogen molecules that serve as antigens or agonists of innate immune receptors to induce host defense and immunity or serve as regulators of host defense and mediators of immune evasion^50,52^. We speculate that the mechanism of camouflaging the virus from immune surveillance might rather trigger an autoimmune response from the host in those instances where the close association of viral and host antigens promotes their crossed-recognition.

### The intra-cytoplasmic route of SARS-COV-2 transmission

Imaging employing the nanometer resolution, lack of coating and practical imaging of samples provided by HeIM suggest that SARS-CoV-2 infected Vero E6 cells can establish connections by TNT. HeIM also confirmed that they could form syncytia and exchange EV. These three features co-exist together in space and time. In all three processes, there is an exchange of cytoplasmic content between host cells. This may be an alternative route of transmission and infection, clearly distinct from the well-known, conventional extra-cytoplasmic ACE2 (or other receptor-mediated) docking mechanism. Even if the close phylogenetic relationship between SARS-CoV and SARS-CoV-2 makes it reasonable to translate much of our knowledge of SARS-CoV to SARS-CoV-2, we should focus on the differences between SARS-CoV and SARS-CoV-2 to explain divergent clinical patterns of disease caused by these two viruses. We already cited the fusogenic potential specific to the SARS-CoV-2 spike protein, but not the SARS-CoV spike protein^46^. Notably, SARS-CoV-2 has a lower density of spikes and can produce a high number of defective copies (with little or no spikes) released outside the infected cell^61,62^. These observations seem like strong, even if indirect, evidence that an alternative route of viral propagation must be in place for SARS-CoV-2.

The intra-cytoplasmic route can hide the virus from the host immune surveillance and potential anti-viral response, which are based on the detection of the extracellular virions. In the intra-cytoplasmic route, naïve cells could be infected by viral mRNA, transmitted free, or in the form of cargo in micro-vesicles. Defective virions, presenting little or no spikes, which would be ineffective in infecting other cells via the extra-cytoplasmic route, might become effective when transmitted via the intra-cytoplasmic route. Host defenses based on a humoral or cellular immune response will be mostly ineffective against this intra-cytoplasmic spreading of SARS-CoV-2. More than two decades of failure to realize a protective vaccine for HIV highlights the need for a better understanding of the viral immune evasion mechanisms^52^; possibly, a similar case is mounting for SARS-CoV-2.

### Broader implications

The vast majority of diseases, including COVID-19, are complex in their presentation and may have multiple stages. Each stage may have a specific/targeted and/or a generic/multiple-targets therapeutical approach (many forms of cancer fit in this description). We consider that our findings may have a clinical relevance as the possibility of an alternative route of transmission (in respect to the ACE2-docking) means that this route can require specific therapeutic strategies. Specific drugs that target the mechanisms of this intra-cytoplasmic route (TNT, Fusion, EV) will be, theoretically, promising candidates to treat COVID19. However, the analysis of the spectrum of possible therapeutic molecules that impair the intracytoplasmic route of viral transmission is beyond the scope of this paper. We are not suggesting or promoting any specific drug.

We reason that if drugs already in use, and known to target the mechanisms of this alternative route (either TNT formation, or cell Fusion, or EV metabolism), are effective in the contrast of SARS-CoV-2 infection, this could be taken as indirect evidence that the intra-cytoplasmic route plays a role in the infection. A simple search in the literature showed us that several drugs interfering with the TNT, Fusion or EV have been empirically tested already. We provide some examples. (A) In targeting TNT, the proven clinical efficacy of Colchicine, a weel-known microtubule inhibitor that might interfere with TNT formation^63,64^, can be suggestive evidence of the role of TNT in COVID19 pathogenesis. (B) Several drugs that suppress cell fusion have been tested in vitro^47^; among them, Niclosamide was effective in cell protection against virus-induced cell death^45,47^. (C) Drugs that interfere with EV machinery at large have been tested even before the COVID-19 pandemic, during the SARS and MERS emergencies. Chloroquine is well-known for elevating the pH in endosomal vesicles; apart from its known efficacy in Malaria, it has been shown to be effective against SARS-CoV infection in vitro^65^ and in COVID-19 cases^66^. It is important to state that the alternative intra-cytoplasmic route also suggests a modality of administration, in the sense that a candidate drug will likely need to be administered during the early stages of SARS-CoV-2 infection, when TNT formation, cell fusion and EV release occur. No prevention therapy (when there are no TNT formation, cell fusion or EV release), or later stage therapy (when other mechanisms, like hyper-inflammation or autoimmunity, are in place) can be suitable.

Limitations in our study include the absence of wider use of specific markers to track the viral transmission and the lack of usage of primary human cells to confirm our findings. However, observations reported here are actually stimuli for planning new studies, not only by us but also by the larger scientific community. Having highlighted the presence of a potential intracytoplasmic route for SARS-COV-2 transmission and infection can provide a pathophysiological explanation for how empirical therapies already in use may work. It can also promote efforts towards the identification of new therapeutic agents targeted to this route. It can address the question, that we reported in the Introduction, as to the cause of immune escape and renewed disease progression in fully vaccinated individuals.

## Methods

### Helium-ion microscopy

The principle of HeIM operation is very similar to SEM, except that the HeIM utilizes a beam of positively charged helium ions (He+) instead of negatively charged electrons to excite and detect secondary electrons from the sample surface^67^. Due to the high brightness and low energy spread of its atomically sharp gas-field ion source, the smallest attainable focused spot size is about 0.3 nm. With its significantly smaller convergence angle compared to SEM, HeIM achieves a much larger depth of field^67^, which is particularly useful for imaging three-dimensional structures. Due to their higher mass, He+ ions do not spread as much as electrons, resulting in a smaller escape volume of the secondary electrons and a higher surface resolution of the HeIM than SEM. A further benefit of HeIM is its charge compensation capability during secondary electron detection. In the HeIM, charges that accumulate on insulating samples are positive; so, a low-energy electron “flood gun”, which irradiates the sample with a diffuse beam of electrons, is used to achieve charge neutralization. This method eliminates the need for a conductive coating of samples and allows for direct visualization of their morphology at a nanoscale level^22^.

We used a Zeiss Orion Plus helium ion microscope (Zeiss, Peabody MA, USA) at an acceleration voltage of 30 kV and chamber base pressure of 3 × 10^–7^ Torr. The typical Helium beam had a 0.7 pA current which was achieved by varying the spot control between 5 and 6 to adjust lens 1, coupled with using 10 µm aperture. The sample stage was tilted by 10°, and the working distance was kept in the 8–10 mm range. Since all the studied samples were nonconductive, the electron flood gun was used to eliminate charging effects, with an electron flood time of 10 µs and He^+^ beam dwell time of 0.2 µs.

### Vero E6 cell culture and SARS-CoV-2 infection

The African green monkey kidney epithelial cell line (Vero E6) has been used extensively for SARS-CoV research in cell culture-based infection models. The lineage was developed in 1962 by Yasumura and Kawakita at the Chiba University in Japan deriving cells from a female of Chlorocebus sabaeus^5^. Vero E6 cells support SARS-CoV-2 replication in culture, while many more cell lines have been reported to be refractory to SARS-CoV-2 infection^68^. The close phylogenetic relationship between SARS-CoV and SARS-CoV-2, the abundantly expressed ACE2 on the Vero E6 membrane, and their characteristic of being deficient in interferon-alpha or beta^69^, could explain their susceptibility to SARS-CoV-2 infection.

All the work involving infectious SARS-CoV-2 was performed in the biosafety level 3 facilities at Rutgers University. The SARS-CoV-2 expressing mNeonGreen was provided by Dr. Theresa Chang (Rutgers University) and Dr. Pei-Yong Shi (University of Texas)^70^. Vero E6 cells were grown in Dulbecco’s modified essential medium (DMEM) (Ca# D6429, Sigma-Aldrich, USA) containing 10% fetal bovine sera (FBS) (Sigma-Aldrich, USA). For SARS-CoV-2 infection, 0.3 × 10^6^ Vero E6 cells were seeded onto a 13 mm diameter glass coverslips with carved reference frames, in a six-well cell-culture plate with 2 mL of DMEM + FBS media. At 18–24 h post-seeding, the spent media was aspirated. The cells were infected with SARS-CoV-2 expressing mNeonGreen at a multiplicity of infection (MOI) of 0.1 (i.e., 1 viral particle for 10 host cells) in 400 µL of FBS-free DMEM. The plates were incubated at 37 °C for 1 h with intermittent rocking for infection. Then the wells were replenished with 2 mL of DMEM + FBS. At 24 h post-infection, the cell culture supernatants were removed and the cells were fixed with 4% paraformaldehyde (Cat# 19943-k2, Thermo Scientific, USA) as described previously^71^.

### Correlative microscopy

Reference frames carved on the glass coverslips allowed us to perform correlative HeIM-Fluorescence-Phase Contrast microscopy (Zeiss Axio Observer, Zeiss Jena D). The ability to practically identify and study the same cell in these three different imaging modalities was extremely useful, as we could merge information specifically provided by each of them. As HeIM does not require any electroconductive coating, samples imaged by HeIM could be re-imaged with phase contrast or fluorescence microscopy, as needed (an option which is not available by using SEM because of the coating). The routine sequence of imaging usually started with Phase Contrast microscopy, followed by Fluorescence microscopy. Vero E6 cells infected with SARS-CoV-2 were identified by the green fluorescence of the mNeonGreen protein expressed by the virus intracellularly. We were able to detect the green fluorescence with a filter set at excitation peak at 488 nm and an emission peak at 509 nm, with a Zeiss Axiocam 305 CCD camera and Zeiss Zen Blue 2.6 software (the optimal excitation and emission peaks for mNeonGreen protein are at 506 nm and 517 nm, respectively^72^). Samples imaged in phase contrast and fluorescence microscopy were in the hydrated state in the culture well. They were dehydrated and put under high vacuum at 3 × 10^–7^ Torr for HeIM imaging. If a new phase-contrast and/or fluorescence microscopy imaging was required, samples were eventually re-hydrated without any damage to the cellular structures.

## Supplementary Information

Supplementary Information.

## Data Availability

Data will be available on-line at the RUcore (Rutgers University Community Repository).
