# Supplementary material for: An intra-cytoplasmic route for SARS-CoV-2 transmission unveiled by Helium-ion microscopy
Source: Sci Rep. 2022 Mar 8;12:3794. doi: 10.1038/s41598-022-07867-0 (PMC8904465; doi:10.1038/s41598-022-07867-0)
Supplement: Supplementary file 1 — Supplementary Information. [file 41598_2022_7867_MOESM1_ESM.docx]

**Supplementary Information**

**
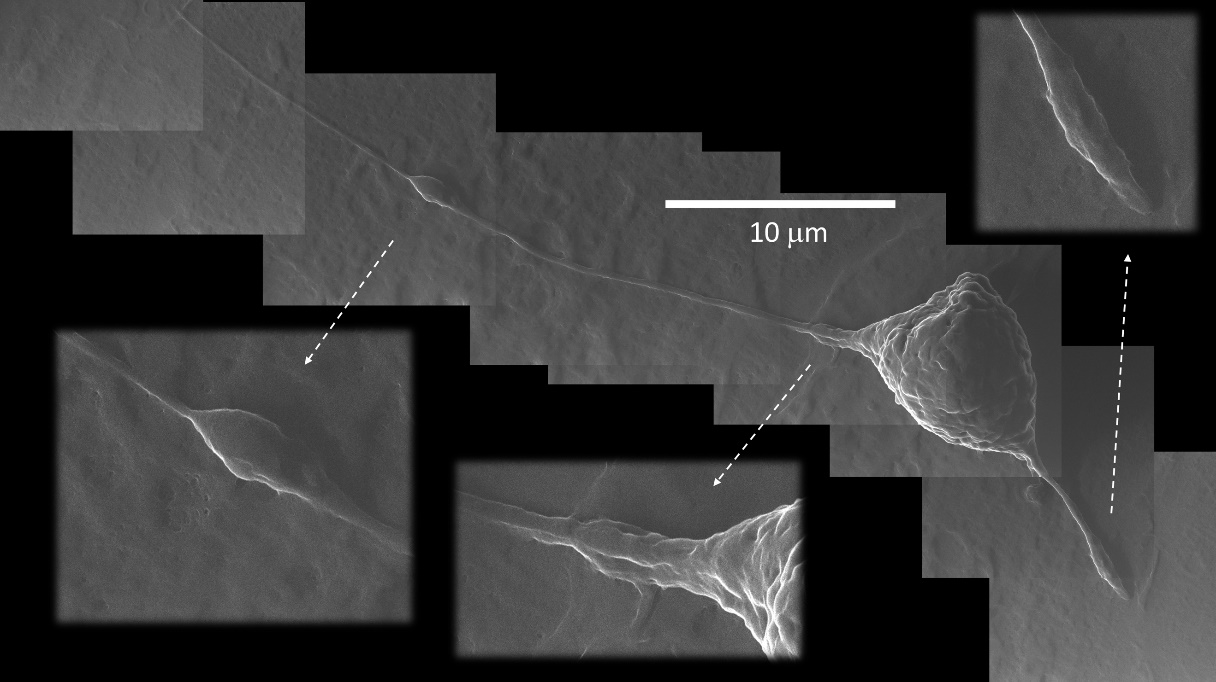
**

**Supplemenatry Information S1: Details of a polarized infected cell.** Cell #3 in figure 2 is visualized in a mosaic of photomicrographs taken at 27,000X.

**
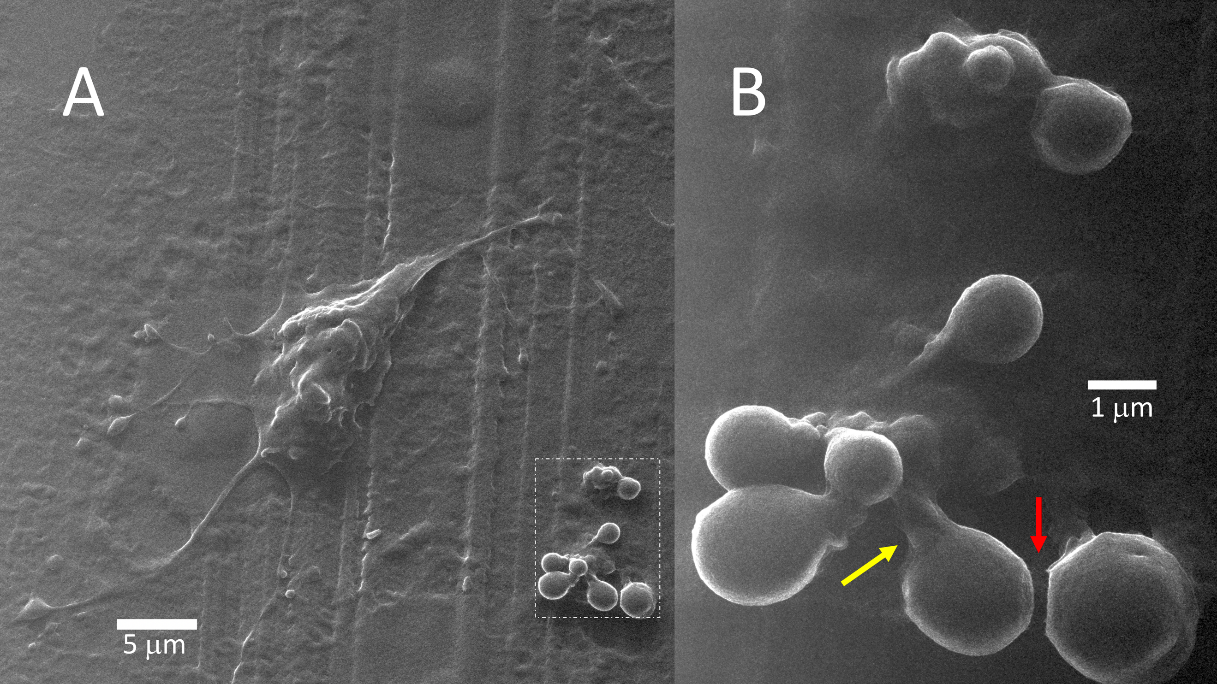
**

**Supplementary Information S2: Apoptosis of SARS-CoV-2 infected host cell.** Budding TNT with growth cones (A) may resemble budding apoptotic bodies captured just before leaving a disintegrating cell (B). Apoptotic bodies in B show a clear stalk (yellow arrow) and images suggesting a close membrane apposition (red arrow; detachment possibly due to dehydration). The parallel lines are the result of glass carving for reference frames. (magnification in A 5,400X; in B 27,000X)


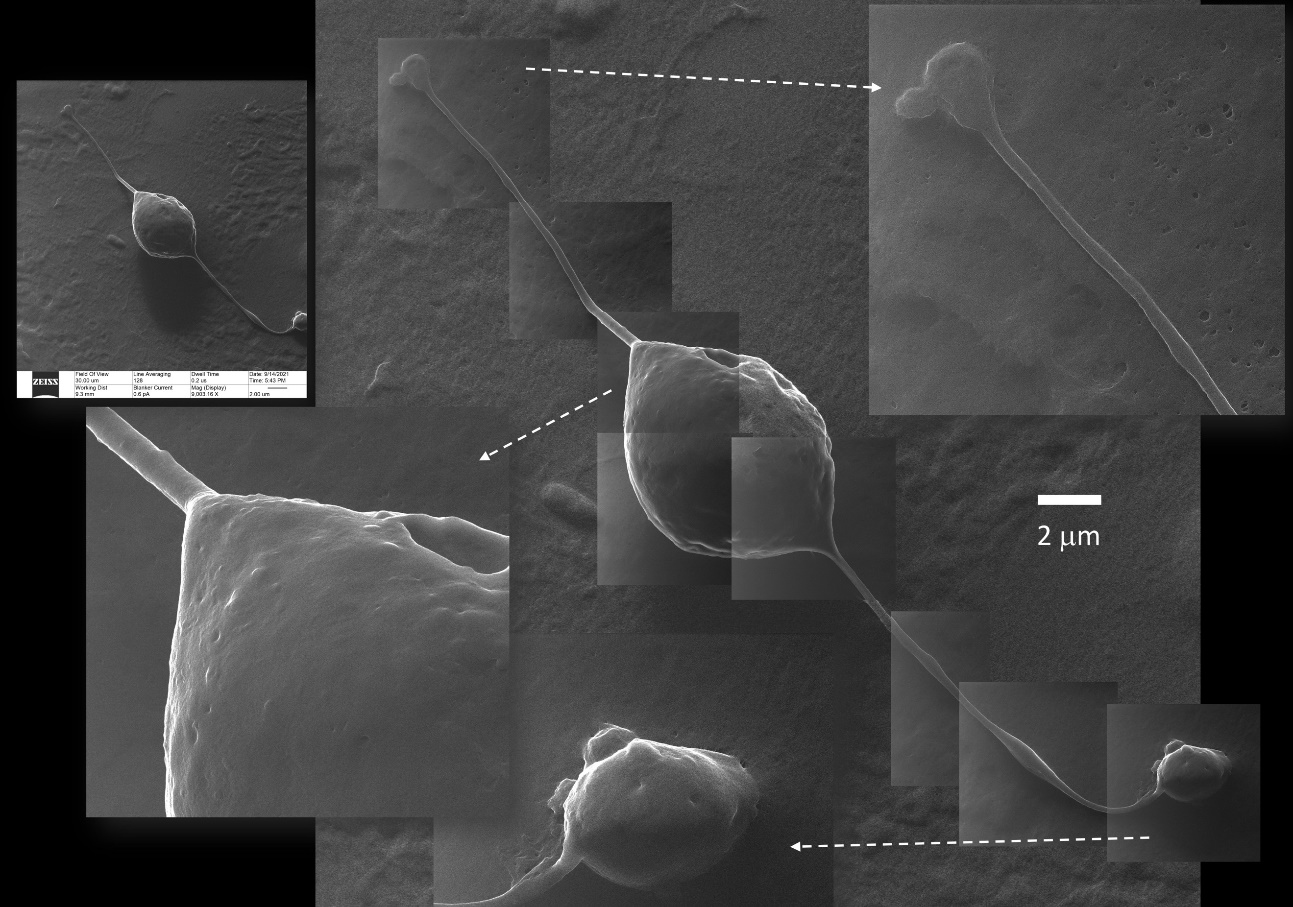


**Supplementary Information S3: A solitary bipolar cell.** This bipolar cell bulged and detached from the floor. Phase-contrast microscopy showed that it changed its position apart from its growth cone attachment. Fluorescence microscopy showed no signal, denoting that it was dead at the time of fixation. (magnification inset and background 9,000X; details 54,000X)
